# In-Hospital Outcomes of Acute Ischemic Stroke in Patients With Hypertrophic Cardiomyopathy

**DOI:** 10.1016/j.mayocpiqo.2022.12.003

**Published:** 2022-12-30

**Authors:** Daokun Sun, Hartzell V. Schaff, Rick A. Nishimura, Jeffrey B. Geske, Joseph A. Dearani, Steve R. Ommen

**Affiliations:** a Department of Cardiovascular Surgery, Mayo Clinic, Rochester, MN; b Department of Cardiovascular Medicine, Mayo Clinic, Rochester, MN

**Keywords:** AF, atrial fibrillation, HCM, hypertrophic cardiomyopathy, ICD-10, International Statistical Classification of Diseases and Related Health Problems, Tenth Revision, NIS, National Inpatient Sample

## Abstract

**Objective:**

To investigate the in-hospital outcomes of acute ischemic stroke in patients with hypertrophic cardiomyopathy (HCM).

**Patients and Methods:**

Using weighted discharge data from the National Inpatient Sample, we identified 5804 nonelective hospitalizations for ischemic stroke in adult patients with HCM between 2011 and 2017. For comparison, 58,179 hospitalizations for ischemic stroke in adult patients without HCM were selected as controls using the simple random sampling method.

**Results:**

Compared with the patients without HCM, those with HCM had a higher prevalence of hyperlipidemia (62.4% vs 57.5%, respectively, *P*<.001) and chronic heart failure (25.4% vs 13.6%, respectively, *P*<.001) but a lower prevalence of diabetes (28.2% vs 34.9%, respectively, *P*<.001) and hypertension (42.9% vs 53.4%, respectively, *P*<.001). Atrial fibrillation was documented in 45.1% (n=2617) of the patients with HCM. However, only 28.0% (n=733) of these patients had long-term use of anticoagulants. The in-hospital death rate among the patients with HCM was 6.3% (n=368), which was significantly higher than that in the patients without HCM (4.1%, *P*<.001). Having HCM (odds ratio [OR], 1.35; *P*<.001), atrial fibrillation (OR, 2.08; *P*<.001), and chronic heart failure (OR, 1.65; *P*<.001) were significant predictors of in-hospital death. In patients with HCM who were discharged alive, 50.0% were transferred to skilled nursing facilities compared with 45.3% of those without HCM (*P*<.001).

**Conclusion:**

The prognosis of acute ischemic stroke is worse in patients with HCM than in those without HCM. These findings emphasize the importance of aggressive treatment of predisposing factors for stroke in patients with HCM, especially atrial fibrillation.

Ischemic stroke is a serious thromboembolic complication of hypertrophic cardiomyopathy (HCM), which is largely attributable to atrial fibrillation (AF).^1^^,^^2^ In a previous cohort study, Maron et al^3^ reported that during an average duration of 7 years of follow-up, ischemic stroke occurred in 20% of patients with AF and 0.7% of those without AF. More importantly, 89% of patients with HCM who experienced at least 1 episode of ischemic stroke had AF at baseline. In the general population, the estimated in-hospital mortality rate of ischemic stroke is 4.9%, and patients with AF have a 30%-70% increased risk of in-hospital death after adjusting for other risk factors.^4^ However, despite the high prevalence of AF in patients with HCM, little is known about the short-term prognosis of acute ischemic stroke in patients with HCM. In this study, we analyzed data from a large all-payer inpatient care database in the United States to describe the clinical profile and outcomes of hospitalization for acute ischemic stroke in patients with HCM.

## Methods

### Patient Population

Discharge data from the National Inpatient Sample (NIS) database were queried for this study. The NIS is a large administrative database in the United States that represents approximately 20% of hospital discharges from community hospitals participating in the Healthcare Cost and Utilization Project.^5^ The NIS included all discharges annually from 20% of sampled hospitals before 2012. The sampling strategy was modified in 2012, and 20% of sampled discharges from all hospitals participating in the Healthcare Cost and Utilization Project were included in the NIS annually since then. The database provides discharge-level sampling weight to produce national estimates. We identified nonelective admissions of adult patients (aged ≥18 years) with a primary diagnosis of acute ischemic stroke. The hospital admissions were classified into the HCM-related (case) or non-HCM–related (control) group on the basis of secondary diagnoses. For comparison of clinical features between patients with HCM and those without HCM, a subset of the hospital admissions of the patients without HCM was selected using the simple random sampling method, which resulted in a case-to-control ratio of 1:10 in the unweighted sample. Discharge diagnoses and baseline comorbidities were identified using International Classification of Disease, Ninth Revision, and International Statistical Classification of Diseases and Related Health Problems, Tenth Revision (ICD-10), codes (Supplemental Table, available online at http://www.mcpiqojournal.org). Atrial fibrillation documented using ICD-10 codes was further classified as paroxysmal, persistent or chronic, or unspecified AF.

### Statistical Analyses

Descriptive statistics are reported using median (interquartile range [IQR]) and count (percentile) for continuous and categorical variables, respectively. To compare clinical features between patients with HCM and those without HCM, the Mann-Whitney U test and Chi-square test (or Fisher exact test) were used for univariable analysis. Independent predictors of in-hospital death were identified using a multivariable logistic regression model. All analyses were performed within the weighted discharge data using R, version 4.0.2 (R Foundation for Statistical Computing), and SPSS Statistics, version 28.0 (IBM Corp).

## Results

### Baseline Characteristics

We analyzed 5812 weighted hospitalizations for acute ischemic stroke between 2011 and 2017 in patients with HCM, which were compared with 58,179 weighted hospital admissions in patients without HCM (Table 1). The median (IQR) age of the patients with HCM (72 [62-82] years) was similar to that of those without HCM (72 [61-82] years, *P*=.543); however, women comprised a higher proportion of the study population with HCM (60.3% vs 50.3%, *P*<.001). Compared with the non-HCM group, patients with HCM had a lower prevalence of type 2 diabetes (28.2% vs 34.9%, *P*<.001) and systemic hypertension (42.9% vs 53.4%, *P*<.001) but a higher prevalence of hyperlipidemia (62.4% vs 57.5%, *P*<.001), chronic heart failure (25.4% vs 13.6%, *P*<.001), and a history of previous myocardial infarction (9.6% vs 7.3%, *P*<.001).

**Table 1:** Baseline Characteristics Stratified by the Presence or Absence of Hypertrophic Cardiomyopathy^a^

Variable	HCM (n=5812)	Non-HCM (n=58,179)	*P* value
Age (y)	72 (62-82)	72 (61-82)	.54
Female	3502 (60.3%)	29,042 (50.3%)	<.001
Obesity	674 (11.6%)	6356 (11.0%)	.16
Type 2 diabetes	1636 (28.2%)	20,158 (34.9%)	<.001
Systemic hypertension	2490 (42.9%)	30,865 (53.4%)	<.001
Hyperlipidemia	3621 (62.4%)	33,242 (57.5%)	<.001
Chronic heart failure	1472 (25.4%)	7857 (13.6%)	<.001
History of previous myocardial infarction	558 (9.6%)	4231 (7.3%)	<.001
Atrial fibrillation	2617 (45.1%)	14,512 (25.1%)	<.001
Long-term (current) use of anticoagulants	848 (14.6%)	4425 (7.7%)	<.001

HCM, hypertrophic cardiomyopathy.

a Values represent the median (interquartile range) for continuous variables and count (%) for categorical variables.

Long-term (current) use of anticoagulants at the time of hospital admission for ischemic stroke was documented in 14.6% (n=848) of the patients with HCM and 7.7% (n=4425) of those without HCM (*P*<.001). Atrial fibrillation was present in 45.1% (n=2617) and 25.1% (n=14,512) of hospitalizations in the HCM and non-HCM groups (*P*<.001), respectively, and long-term (current) use of anticoagulants was documented in 28.0% (n=733) and 21.7% (n=3152) of these patients with AF, respectively (*P*<.001). In a subset of 5875 hospital admissions of patients with AF that were coded using ICD-10 codes, paroxysmal AF and persistent or chronic AF were reported in 43.0% (n=460) and 23.4% (n=250) of the patients with HCM, respectively, and in 34.2% (n=1645) and 24.9% (n=1195) of those without HCM, respectively (*P*<.001).

### In-hospital Outcomes

Tissue plasminogen activator was administered at the admitting hospital in 7.3% (n=425) of the patients with HCM and 7.5% (n=4313) of those without HCM (*P*=.69) (Table 2). Compared with those in the non-HCM group, patients in the HCM group were more likely to undergo endovascular thrombectomy (4.5% vs 2.1%, respectively, *P*<.001). The patients with HCM were also more likely to receive tissue plasminogen activator at a different facility within 24 hours before being transferred to the admitting hospital (3.6% vs 2.3%, *P*<.001).

**Table 2:** Stratified In-hospital Outcomes in Patients With and Without Hypertrophic Cardiomyopathy^a^

Variable	HCM (n=5812)	Non-HCM (n=58,179)	*P* value
Treatment of stroke			
Tissue plasminogen activator	425 (7.3%)	4313 (7.5%)	.69
Endovascular thrombectomy	260 (4.5%)	1187 (2.1%)	<.001
Drip-and-ship thrombolytic therapy	210 (3.6%)	1340 (2.3%)	<.001
Length of stay (d)	4 (2-7)	3 (2-6)	<.001
Total charge ($)	36,723 (21,491-66,840)	32,888 (20,130-56,590)	<.001
In-hospital death	368 (6.3%)	2344 (4.1%)	<.001
Transfer to SNF, ICF, and others	2717 (50%)	25,082 (45.3%)	<.001

HCM, hypertrophic cardiomyopathy; ICF, intermediate care facility; SNF, skilled nursing facility.

a Values represent the median (interquartile range) for continuous variables and count (%) for categorical variables.

**Figure 1:**
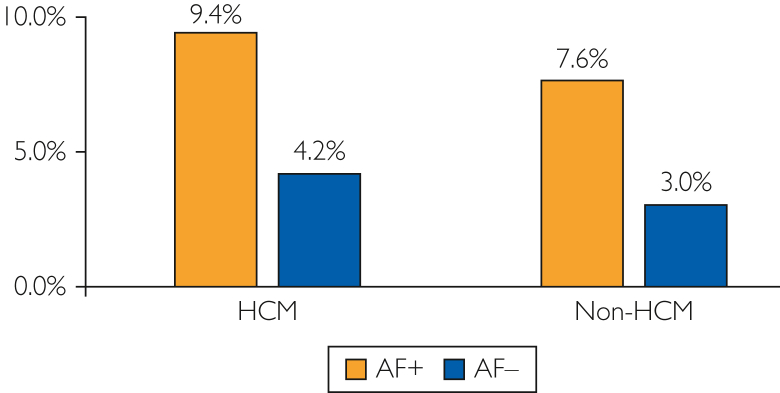
In-hospital death rates of ischemic stroke stratified by the presence of atrial fibrillation among patients with and without hypertrophic cardiomyopathy. AF, atrial fibrillation; HCM, hypertrophic cardiomyopathy.

**Figure 2:**
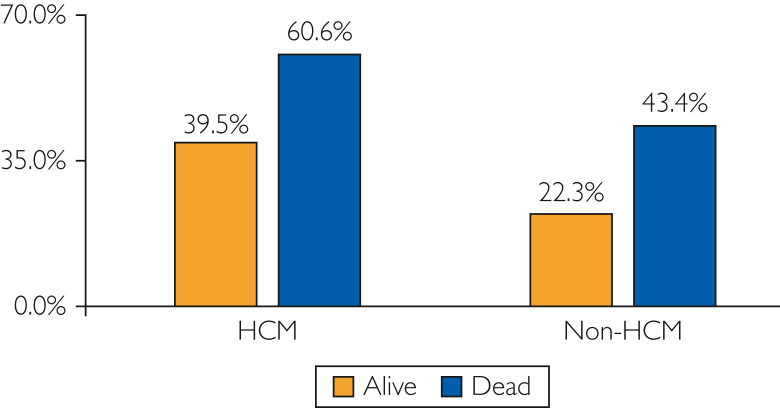
Prevalence of atrial fibrillation stratified by vital status at hospital dismissal in patients with and without hypertrophic cardiomyopathy. HCM, hypertrophic cardiomyopathy.

The median (IQR) length of stay was longer in the HCM group (4 [2-7] days) than in the non-HCM group (3 [2-6] days, *P*<.001). There were 2712 deaths during the hospital stay; 368 occurred in the HCM group and 2344 in the non-HCM group (*P*<.001) (Figures 1 and 2). The total charge of hospitalizations for acute ischemic stroke was $36,723 ($21,491-$66,840) in the patients with HCM, which was higher than that in those without HCM ($32,888 [$20,130-$56,590], *P*<.001). After hospital dismissal, 50.0% of the patients with HCM and 45.3% of those without HCM discharged alive were transferred for rehabilitation to skilled nursing facilities or intermediate care facilities rather than to other short-term hospitals or home health care for poststroke care (*P*<.001).

### Predictors of In-hospital Death

Unadjusted and adjusted associations of in-hospital death with baseline characteristics are shown in Table 3. In addition to the disease status of HCM, all baseline demographic variables were included in the multivariate model to identify independent predictors of in-hospital death. We found that having AF was significantly associated with a higher risk of in-hospital mortality (*P*<.001), whereas long-term (current) use of anticoagulants was associated with a reduced risk of death (*P*<.001). After adjusting for all baseline comorbidities, the diagnosis of HCM remained a significant risk factor for in-hospital death after ischemic stroke.

**Table 3:** Unadjusted and Adjusted Associations of In-hospital Mortality With Baseline Characteristics

Variable	Odds ratio^a^	*P* value	Odds ratio^b^	*P* value
Age (y)	1.03	<.001	1.02	<.001
Female	1.41	<.001	1.15	<.001
Chronic heart failure	2.22	<.001	1.65	<.001
Atrial fibrillation	2.69	<.001	2.08	<.001
Long-term (current) use of anticoagulants	0.55^c^	<.001	0.62	<.001
Hypertrophic cardiomyopathy	1.60	<.001	1.35	<.001

a Univariate model.

b Multivariate model adjusting for age, sex, obesity, type 2 diabetes, systemic hypertension, hyperlipidemia, history of myocardial infarction, chronic heart failure, atrial fibrillation, long-term (current) use of anticoagulants, and hypertrophic cardiomyopathy.

c Analysis was performed with adjustment for the status of atrial fibrillation.

## Discussion

Ischemic stroke, which accounts for 87% of all strokes,^6^ is one of the major causes of death in the general population. In patients with HCM, the estimated incidence of stroke and/or systemic embolic events is between 0.8% and 1.0% per year.^3^^,^^7^ The risk of ischemic stroke is increased 5 fold in the presence of AF,^8^ and arrhythmia is associated with not only reduced survival but also greater residual neurologic disability among stroke survivors.^9^^,^^10^ The prevalence of AF in patients with HCM is approximately 20%.^11^^,^^12^ Although the prevalence of AF increases with age,^13^ AF is an important clinical problem in all adults with HCM, and the management of AF is crucial to the prevention of ischemic stroke in patients with HCM. In this study, we compared the national prevalence of AF and other cardiovascular risk factors between patients with HCM who experienced acute ischemic stroke and those without HCM. We found that compared with patients without HCM, those with HCM who were hospitalized for acute ischemic stroke were 80% more likely to have AF, and their chance of having a fatal stroke during the hospital stay was 57% greater than the others’. In addition, stroke survivors with HCM more often required poststroke rehabilitation at skilled nursing facilities or intermediate care facilities.

As we have shown in the present study, the in-hospital death rate after ischemic stroke is 6.3% in patients with HCM and 9.4% in patients with HCM and AF. Indeed, these figures underestimate the overall impact of ischemic stroke on survival given that 64% of stroke-related deaths occur outside of acute care hospitals.^14^ Previously, in a community-based cohort with HCM and AF, Olivotto et al^13^ reported incident strokes in 23 of 107 (21%) middle-aged patients (mean age, 50 years) during an average duration of 13 years of follow-up; 35% of these 23 patients experienced stroke-related death. Given that both the incidence of stroke and mortality increase with age,^14^ the overall risks of ischemic stroke and stroke-related death in patients with HCM and AF may be even higher than that reported.

Systemic anticoagulation reduces the risk of stroke in the general population,^15^^,^^16^ and the benefits of oral anticoagulants have also been confirmed in patients with HCM. Among 900 patients with HCM managed at experienced HCM centers, Maron et al^3^ reported that in patients without long-term anticoagulation therapy for paroxysmal or chronic AF, the occurrence of stroke and peripheral arterial embolization is 72% higher than that in patients with anticoagulation therapy with warfarin (18%). Importantly, the use of anticoagulants was reported in only 29% of patients who experienced stroke or embolic events, and this underuse of anticoagulants is similar to the findings in the present study among patients with HCM and AF who experienced stroke.^3^ Current guidelines recommend the consideration of therapeutic anticoagulation in all patients with HCM and AF, regardless of other risk stratification tools such as CHADSVasc.^2^

Using the ICD-10 codes, we found that persistent or chronic AF was documented in 23.4% of the patients with HCM and ischemic stroke, although the type of AF was unspecified in another 33.6%. In contrast, Maron et al^3^ reported that 62% of patients with HCM who were identified as having AF and experienced stroke or embolic events had chronic AF at baseline. Indeed, the detection rate of AF varies depending on the frequency, modality, and duration of surveillance.^17^ However, the lower proportion of patients with chronic AF in the present study may be explained by the inclusion of newly diagnosed AF during hospitalization for ischemic stroke. Although administrative data do not distinguish pre-existing AF from new-onset AF, the prevalence ratio of persistent or chronic AF vs that of paroxysmal AF suggests an increased burden of AF during hospitalization for ischemic stroke.

### Limitations

The limitations of this study include the lack of granular information of individual patients, especially those with HCM, and the inability of the data to identify patients with a history of stroke or transient ischemic attack before the index event. The present investigation was a cross-sectional study, which could not address the causal association of ischemic stroke with traditional cardiovascular risk factors. The study findings are also limited by the accuracy of data coding in administrative data sets. Further, we only included hospitalizations with acute ischemic stroke coded as the primary diagnosis, which underestimates the overall prevalence of hospitalization for ischemic stroke.

## Conclusion

In the present study, we found that the in-hospital mortality rate of acute ischemic stroke increased in the patients with HCM. This may be explained, in part, by the high prevalence of AF as well as other risk factors related to cardiomyopathy and chronic heart failure. The cause of this association is unclear but may be related to arrhythmia and/or underlying hemodynamic abnormalities not captured in the data set. Further, the data suggest that systemic anticoagulation is underutilized in patients with HCM and AF despite the current guideline recommendations. These findings emphasize the importance of aggressive treatment of predisposing factors for stroke in patients with HCM, especially for the management of AF. Future studies are needed to investigate the benefits of early treatment of HCM and HCM-related complications in reducing the risk of ischemic stroke and improving the outcomes of acute ischemic stroke in patients with HCM.

## Potential Competing Interests

The authors report no competing interests.
