# Supplementary material for: In-Hospital Outcomes of Acute Ischemic Stroke in Patients With Hypertrophic Cardiomyopathy
Source: Mayo Clin Proc Innov Qual Outcomes. 2022 Dec 30;7(1):45–50. doi: 10.1016/j.mayocpiqo.2022.12.003 (PMC9811196; doi:10.1016/j.mayocpiqo.2022.12.003)
Supplement: Supplementary table [file mmc1.docx]

**Supplementary Table 1.** International Classification of Disease, Ninth Revision (ICD-9) and Tenth Revision (ICD-10) codes for diagnosis

| **Diagnosis** | **ICD-9/ICD-10 code(s)** |
| --- | --- |
| Ischemic stroke | 43301, 43311, 43321, 43331, 43381, 43391, 43401, 43411, 43491, I6300, I63011, I63012, I63013, I63019, I6302, I63031, I63032, I63033, I63039, I6309, I6310, I63111, I63112, I63113, I63119, I6312, I63131, I63132, I63133, I63139, I6319, I6320, I63211, I63212, I63213, I63219, I6322, I63231, I63232, I63233, I63239, I6329, I6330, I63311, I63312, I63313, I63319, I63321, I63322, I63323, I63329, I63331, I63332, I63333, I63339, I63341, I63342, I63343, I63349, I6339, I6340, I63411, I63412, I63413, I63419, I63421, I63422, I63423, I63429, I63431, I63432, I63433, I63439, I63441, I63442, I63443, I63449, I6349, I6350, I63511, I63512, I63513, I63519, I63521, I63522, I63523, I63529, I63531, I63532, I63533, I63539, I63541, I63542, I63543, I63549, I6359, I636, I6381, I6389, I639 |
| Hypertrophic cardiomyopathy | 42511, 42518, I421, I422 |
| Atrial fibrillation | 42731, I480, I4811, I4819, I481, I482, I4820, I4821, I4891 |
| Long-term (current) use of anticoagulants | V5861, Z7901 |
| Obesity | 27800, 27801, V8530, V8531, V8532, V8533, V8534, V8535, V8536, V8537, V8538, V8539, V8541, V8542, V8543, V8544, V8545, E669, E6601, Z6830, Z6831, Z6832, Z6833, Z6834, Z6835, Z6836, Z6837, Z6838, Z6839, Z6841, Z6842, Z6843, Z6844, Z6845 |
| History of previous myocardial infarction | 412, I252 |
| Type 2 diabetes | 25000, 25002, 25040, 25050, 25060, 25062, 25080, 25090, 3559, 3572, 36201, E119, E1140, E1169, E1165, E118, E1141, E1142, E1151 |
| Hyperlipidemia | 2720, 2721, 2722, 2724, E7800, E781, E782, E785 |
| Systemic hypertension | 4010, 4011, 4019, 40200, 40291, 40390 |
| Chronic heart failure | I5022, I5032, I5042, I509, I110, 4280, 42822, 42832, 42842, 4289 |
